# Thermal Transport in Graphene Oxide Films: Theoretical Analysis and Molecular Dynamics Simulation

**DOI:** 10.3390/nano10020285

**Published:** 2020-02-07

**Authors:** Yi Yang, Dan Zhong, Yilun Liu, Donghui Meng, Lina Wang, Ning Wei, Guohua Ren, Rongxin Yan, Yang Kang

**Affiliations:** 1 Key Laboratory of Agricultural Soil and Water Engineering in Arid and Semiarid Areas, Ministry of Education, Northwest A&F (Agriculture and Forestry) University, Yangling 712100, China; yang_yi@nwsuaf.edu.cn (Y.Y.); nwei@nwsuaf.edu.cn (N.W.); 2 Zhuhai Da Hengqin Science and Technology Development Co. Ltd., Hengqin New Area, Zhuhai 519000, China; 3 State Key Laboratory for Strength and Vibration of Mechanical Structures, School of Aerospace, Xi’an Jiaotong University, Xi’an 710049, China; yilunliu@mail.xjtu.edu.cn; 4 Beijing Institute of Spacecraft Environment Engineering, Beijing 100094, China; mengdonghui@126.com (D.M.); wangxiweigood@163.com (L.W.); wqghren@126.com (G.R.); pigsheepdog@126.com (R.Y.)

**Keywords:** graphene-oxide films, thermal conductivity, interfacial thermal conductance, optimal overlap length

## Abstract

As a derivative material of graphene, graphene oxide films hold great promise in thermal management devices. Based on the theory of Fourier formula, we deduce the analytical formula of the thermal conductivity of graphene oxide films. The interlaminar thermal property of graphene oxide films is studied using molecular dynamics simulation. The effect of vacancy defect on the thermal conductance of the interface is considered. The interfacial heat transfer efficiency of graphene oxide films strengthens with the increasing ratio of the vacancy defect. Based on the theoretical model and simulation results, we put forward an optimization model of the graphene oxide film. The optimal structure has the minimum overlap length and the maximum thermal conductivity. An estimated optimal overlap length for the GO (graphene-oxide) films with degree of oxidation 10% and density of vacancy defect 2% is 0.33 μm. Our results can provide effective guidance to the rationally designed defective microstructures on engineering thermal transport processes.

## 1. Introduction

Graphene-oxide (GO) films show fascinating performance and have been widely applied in various fields [1,2,3,4,5,6]. The exfoliated platelets including the individual GO sheet and poor-quality GO films can directly derive from oxidized bulk graphite via the Hummers method [7,8,9,10]. The oxygenated functional groups (epoxy/hydroxyl groups on surface and carboxyl/carbonyl groups on edge) [11], randomly distributed on the carbon backbone of graphene at various degrees of oxidation [12,13], lead to its good dispersibility in water and organic solvents. GO solution also acts as additives and matrix fillers in the nanocomposites to directly enhance thermal conductivities [14]. Strikingly, GO films should have excellent thermal property as the preparation beforehand to obtain graphene-based film [15]. Although the thermal conductivity of GO films is inferior to that of graphene films, due to the influence of functional groups [16], the GO films still attract significant attention mainly because of the facile fabrication and low-cost features, which easily obtained in experiments and practical application. The thermal conductivity of GO films is much higher than that of polymer-based composites [17,18], the current materials for dissipating heat in electronics [19,20,21,22], thermoelectric technology, and heat managements [23,24,25,26,27,28,29]. Therefore, it is necessary to study the thermal properties of GO films to create high-quality heat applications for micro-electronic devices.

**Figure 1:**
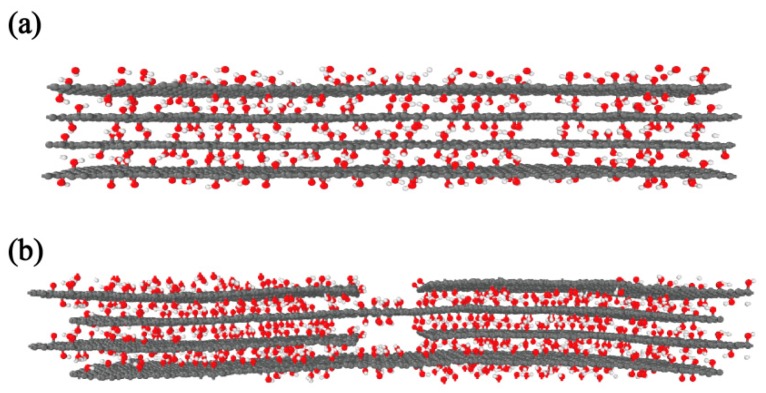
Two stack types of GO films (**a**) a layer-by-layer model (**b**) an overlap model.

The peculiarity and complexity of the flux transfer are a bottleneck for the thermal conductivity thin-film material at atomic scale. Efforts can be made to research the influence factors and thereby improve the quality of such heat transfer efficiency. A multilayered film can be fabricated by a single sheet in a layer-by-layer assembly approach (See in Figure 1a). This nanomaterial with highly anisotropic thermal conductivity (in-plane thermal conductivity $\kappa_{i}$ and out-of-plane thermal conductivity $\kappa_{o}$) can be applied in thermal management of modern electronics. The anisotropy of thermal conductivity of GO films ($\kappa_{i}/\kappa_{o}$ ~ 675) is substantially larger than high-quality bulk graphite ($\kappa_{i}/\kappa_{o}$ ~ 100) [30]. The vacancy defects in the structure lead to different influences in the two directions of thermal conductivity. The in-plane thermal conductivity declines with increasing defect ratio [31,32]. Meanwhile, the out-of-plane thermal conductivity rises with defect ratio because of the strengthening of interlayer attraction [16,33]. Some other measures, which increase the number of layers to enhance van der Waals force [34,35] and the concentration of functional groups to intensify covalent bonding [36], could be taken to improve the out-of-plane thermal conductivity.

Due to the limitation of experimental methods and techniques, the structure with a continuous large individual GO sheet is difficult to synthesize. The GO film structure with gaps is more easily formed (See in Figure 1b) [37]. In this case, we can expand the overlap region [38] and bring in interlayer cross-links [36,39] to increase the heat transfer efficiency in the overlap area of adjacent layers. Up to now, the study on the thermal conductivity of the overlap GO films structure at the atomic scale is still lacking. 

In this article, we highlighted a theoretic formula framework based on Fourier law to decouple the effects of in-plane and out-of-plane on thermal transfer in overlap GO films model. The in-plane thermal property of GO has been studied in our previous work [40]. In this paper, the out-of-plane thermal property of GO is investigated using nonequilibrium molecular dynamics simulations. The effect of degree of oxidation and monatomic vacancy defect on the out-of-plane thermal property is considered. Finally, an optimized overlap length is discussed analytically.

## 2. Model and Methodology

### 2.1. Theoretical Analysis

**Figure 2:**
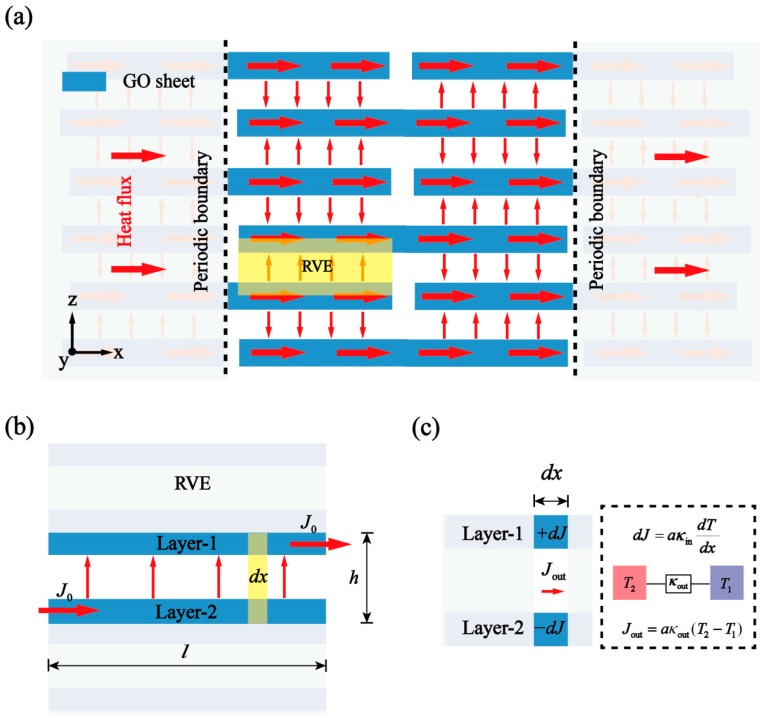
A schematic illustration of the heat-flow model for GO films. (**a**) The overall platelet structure and representative volume element (RVE). (**b**) The heat flow transfer in RVE. The physical parameters and boundary conditions are used in the analytical model. (**c**) The equilibrium relationship between in-plane and out-of-plane heat flow in the infinitesimal area.

In this work, a novel effective theoretical formula of GO films thermal conductivity is deduced based on the Fourier law, systematically considering the heat transfer both in-plane and out-of-plane directions. The derivation of the formula is similar to the previous work on the thermal properties of graphene-based layered materials [39]. Herein, a GO films model with the same overlap length of the interlayer is proposed, as shown in in Figure 2a. A representative volume element (RVE) is shown in Figure 2b. The length and height of the RVE are l and h. Constant heat flux $J_{0}$ flows from the lower layer-2 to the upper layer-1. This means that the reduced heat flow of the lower layer is equal to the increased that of the upper layer. Therefore, the differential equation is deduced on the infinitesimal area (dx) with the Fourier law in Figure 2c, where the increment of heat $J_{1}$ in the upper layer is equal to the inflow heat $J_{\mathrm{out}}$ from other sheets in the structure (Equation (1a)). Correspondingly, the decrement of heat in the lower layer $J_{2}$ is equal to the outflow heat $J_{\mathrm{out}}$ (Equation (1b)). To facilitate calculation, a uniform cross-street area is used in the following derivation, as the thickness of RVE.

(1a) $$\frac{dJ_{1}}{dx}=-J_{\mathrm{out}}=a\kappa_{\mathrm{out}}(T_{2}-T_{1})$$

(1b) $$\frac{dJ_{2}}{dx}=-J_{\mathrm{out}}=a\kappa_{\mathrm{out}}(T_{2}-T_{1})$$

where $T_{1}$, $T_{2}$, and $\kappa_{\mathrm{out}}$ are upper/lower layer temperature and thermal conductance on the infinitesimal area (dx), respectively. a is the cross-sectional area of RVE. The length of the model in the *y*-axis is set to 1, and the value of a is equal to h. The thermal conductivity $\kappa_{\mathrm{in}}$ of the upper layer and the lower layer can be calculated by the following Equation (2).

(2) $$J_{1}=-a\kappa_{\mathrm{in}}\frac{dT_{1}}{dx},J_{2}=-a\kappa_{\mathrm{in}}\frac{dT_{2}}{dx}$$

Bring Equation (2) into Equation (1a,b) and eliminate a. The equation of governing the heat transfer is given as

(3a) $$-\kappa_{\mathrm{in}}\frac{d^{2}T_{1}}{dx^{2}}=\kappa_{\mathrm{out}}(T_{1}-T_{2})$$

(3b) $$-\kappa_{\mathrm{in}}\frac{d^{2}T_{2}}{dx^{2}}=\kappa_{\mathrm{out}}(T_{2}-T_{1})$$

A general solution of Equation (3a,b) is

(4) $$T_{1}+T_{2}=A+Bx$$

(5) $$T_{1}-T_{2}=Ce^{-\lambda x}+De^{\lambda x}$$

λ is given by

(6) $$\lambda =\sqrt{\frac{2\kappa_{\mathrm{out}}}{\kappa_{\mathrm{in}}}}$$

As shown in Figure 2b, the boundary conditions are

(7a) $$\frac{dT_{1}(x=0)}{dx}=0,\frac{dT_{1}(x=l)}{dx}=-\frac{J_{0}}{\kappa_{\mathrm{in}}}$$

(7b) $$\frac{dT_{2}(x=0)}{dx}=-\frac{J_{0}}{\kappa_{\mathrm{in}}},\frac{dT_{2}(x=l)}{dx}=0$$

Using Equation (7) and a few mathematical techniques to substitute for A, B, C, and D in Equation (3a,b) yields

(8a) $$T_{1}=\frac{A}{2}-\frac{J_{0}}{2\kappa_{\mathrm{in}}}x-\frac{J_{0}}{2\lambda \kappa_{\mathrm{in}}}\frac{1+e^{\lambda l}}{e^{\lambda l}-e^{-\lambda l}}e^{-\lambda x}-\frac{J_{0}}{2\lambda \kappa_{\mathrm{in}}}\frac{1+e^{-\lambda l}}{e^{\lambda l}-e^{-\lambda l}}e^{\lambda x}$$

(8b) $$T_{2}=\frac{A}{2}-\frac{J_{0}}{2\kappa_{\mathrm{in}}}x+\frac{J_{0}}{2\lambda \kappa_{\mathrm{in}}}\frac{1+e^{\lambda l}}{e^{\lambda l}-e^{-\lambda l}}e^{-\lambda x}+\frac{J_{0}}{2\lambda \kappa_{\mathrm{in}}}\frac{1+e^{-\lambda l}}{e^{\lambda l}-e^{-\lambda l}}e^{\lambda x}$$

From the above relations, the effective thermal conductivity K of Equation (9) is obtained, that is

(9) $$K=\frac{J_{0}l}{T_{2}(x=0)-T_{1}(x=l)}=\frac{\kappa_{\mathrm{in}}}{\frac{1}{2}+\frac{1}{\lambda l}\frac{2+e^{\lambda l}+e^{-\lambda l}}{e^{\lambda l}-e^{-\lambda l}}}$$

From Equation (9) it can be found that K is determined by the thermal conductivity $\kappa_{\mathrm{in}}$ along the in-plane direction, which has been widely verified that high-quality graphene monoliths can be used to prepare graphene films with super high thermal conductivity [41,42]. λ can be regarded as a structural factor. Once the structure of GO is determined, the degree of oxidation and vacancy ratio are determined, λ is the fixed value. K is only related to the overlap length. The relationship between the $\kappa_{\mathrm{in}}$ in Equation (9) and the thermal conductivity GO $\kappa_{G}$ is

(10) $$2\kappa_{\mathrm{in}}=\kappa_{G}$$

The existence of the constant 2 is due to the periodic conditions of RVE.

(11) $$\kappa_{\mathrm{out}}=\frac{d\kappa_{I}}{dx}$$

$\kappa_{I}$ is referred to as a thermal conductance at interface between GOs. 

### 2.2. Simulation Analysis

**Figure 3:**
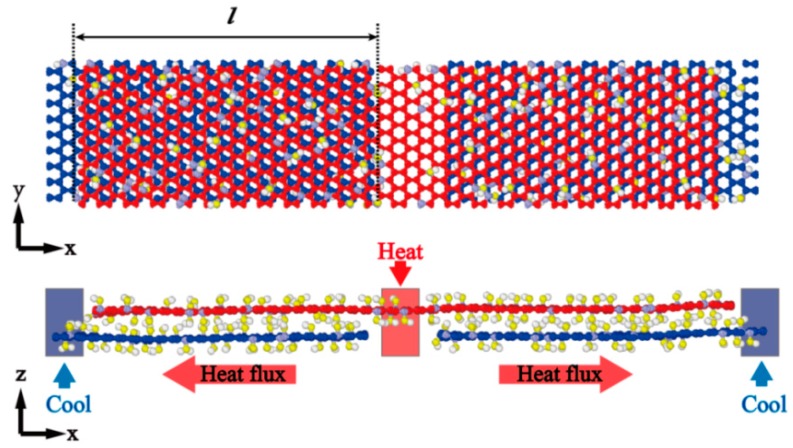
The schematic model for interfacial thermal conductance by the MP algorithm with periodic boundary conditions (*x*- and *y*-axis). The overlap model consists of two GO layers with overlap length (*l*). The heat flow transforms from the upper layer (red) to the lower layer (blue).

In this work, we perform classical molecular dynamics (MD) simulations to study the $\kappa_{I}$ between GO to calculate the $\kappa_{\mathrm{out}}$. The effects of oxygen concentration and vacancy defect ratio on thermal conductance are investigated. To improve the calculation efficiency, we only consider the impact of hydroxyl groups representing oxidation groups due to the little effect on the out-of-plane thermal conductance for different groups [33]. The hydroxyl groups are randomly attached to the carbon atoms on both sides graphene basal plane at different degrees of oxidation from 0% to 10%, while the ratio of removing carbon atoms from GO sheets is from 0% to 2%. The annealing process [30] simulation is from 300 K up to 800 K. Then the system temperature down to 300 K is carried out under the NPT ensemble (constant number of atoms, constant pressure of 1.0 bar) to obtain a stable structure. The atomic coordinates of the pre-formed GO sheet and the periodic dimensions of the simulation cell are optimized using a gradient-based minimization method [43,44] implemented until pressure scaling at zero bar under periodic boundary to relax the structure and reduce the residue stress. The final overlap model of GO films with hydroxyl groups and vacancy defects is shown in Figure 3.

MD simulations are performed using the large-scale atomic/molecular massively parallel simulator (LAMMPS) [45,46]. The all-atom optimized potential for liquid simulations (OPLS-AA) is used for GO, which can capture essential many-body terms in interatomic interactions, including bond stretching, bond angle bending, van der Waals, and electrostatic interactions [47]. This potential is successfully applied in studying water permeation in GO films [48]. The SHAKE algorithm is applied for the stretching terms between oxygen and hydrogen atoms to reduce high-frequency vibrations that require shorter time steps. The interaction between layers includes both van der Waals and electrostatic terms. The former one is described by the Lennard–Jones potential between oxygen and carbon atoms [49]. The van der Waals forces are truncated at 1.2 nm, and the long-range Coulomb interactions are computed by using the particle-particle particle-mesh (PPPM) algorithm [50]. In this work, the thermal conductance is computed by the reverse nonequilibrium molecular dynamics simulations in an NVE ensemble [51]. The key point of the method is to impose a heat flux through the system and to determine the temperature jump from a consequence of the imposed flux.

**Figure 4:**
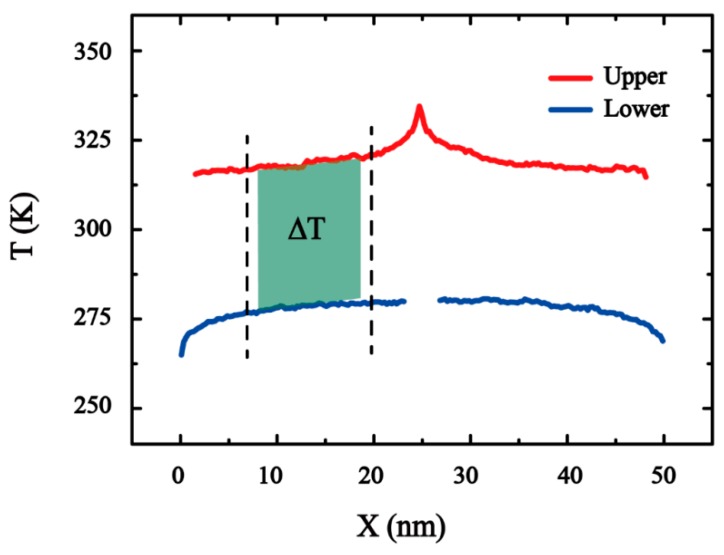
Equilibrium temperature profiles for the overlap model of GO. The temperature difference is obtained by the average value of the common linear portions between the upper and lower layer (dark green area).

The system is equally divided into 100 thin slabs along the heat transfer direction. The heat source and sink slabs are located at the middle and the two ends of the model, respectively. The time step of 0.1 fs is selected for integration on the equations of atomic motion in the simulations. The system reaches the equilibrium state at 300 K in the Nosé–Hoover thermal bath for 0.2 ns. Then, the system exchanges the kinetic energies every 100 time steps between the coldest atom in the heated slab and the hottest atom in the sink source slab for 0.8 ns. The total heat flux J can be obtained from the amount of the injected two slabs by exchanging the kinetic energies.

(12) $$J=\frac{\sum_{N_{\mathrm{tranfers}}}\frac{1}{2}(mv_{h}{}^{2}-mv_{c}{}^{2})}{t_{\mathrm{transfer}}}$$

where $N_{\mathrm{tranfers}}$ is the total number of times of exchanging the kinetic energies; $t_{\mathrm{transfer}}$ is the total time over simulation; m is the mass of each atom; $v_{h}$ and $v_{c}$ are the velocities of the hottest atom in the cold slab and the coldest atom in the hot slab, respectively. When the heat flow reaches the nonequilibrium steady state, the temperature distribution is collected to obtain the temperature jump.

(13) $$T_{i}=\frac{2}{3Nk_{B}}\sum_{j}\frac{p_{j}^{2}}{2m}$$

where $T_{i}$ is the temperature of the *i*-th slab and $p_{j}^{}$ represents the potential energy of the atom j. $k_{B}$ is Boltzmann’s constant. As shown in Figure 4, the temperature profiles are obtained by averaging results of the last 6 million time steps which are collected every 1000 time steps. Small temperature differences increase the calculation time and waste calculation resources, whereas large temperature difference will cause the escape of hydroxyl groups on the surface of graphene oxide. Meanwhile, too much temperature difference between the cold and heat sources will cause phonon scattering, which will affect the accuracy of the calculation results. From other researches, the maximum temperature difference is not more than 100 K [8,35,52,53]. In this paper, we control the heat exchange time to ensure the temperature difference. The existence of temperature jump ΔT across the overlapped portion in Figure 4 (dark green) due to thermal resistance at the overlap area. The interfacial thermal conductance $\kappa_{I}$ of GO is calculated as

(14) $$\kappa_{I}=\frac{J}{2A\Delta T}$$

where A is the cross-section area of the corresponding model and the constant 2 in the denominator arises accounts for the fact that the system is periodicity. The value of $\kappa_{I}$ is obtained by averaging five independent simulation results. Each independent simulation only ensures degree of oxidation constant.

## 3. Result and Discussion

The coupling effect of the hydroxyl-group and vacancy defects on the out-of-plane thermal properties of GO is expressed by interfacial thermal conductance $\kappa_{I}$. First, $\kappa_{I}$ is calculated with a fixed overlap length of 10 nm. We defined a ratio between oxygen and carbon atoms $R_{\mathrm{OH}}$ to describe the degree of oxidation. Also, $R_{V}$ is used as the ratio of vacancy defect in the system. The values of the thermal conductance of the overlap GO model with different $R_{\mathrm{OH}}$ (0–10%) and $R_{V}$ (0–2%) are shown in Table 1. When there is no defect in the structure, the thermal conductance is the minimum ($\kappa_{I}$ = 0.26 × 10^8^ Wm^−2^ K^−1^), the corresponding model is the graphene overlap structure. The effect of thermal transfer between GO strengthened gradually with an increasing degree of oxidation. When $R_{\mathrm{OH}}$ is equal to 10%, the $\kappa_{I}$ is 1.68 × 10^8^ Wm^−2^ K^−1^. $\kappa_{I}$ shows similar trends with the increase of vacancy defect ratio. The maximum $\kappa_{I}$ of the structure is 2.33 × 10^8^ Wm^−2^ K^−1^, whereas both the degree of oxidation and ratio of the vacancy defect are the maximum ($R_{\mathrm{OH}}$ = 10%, $R_{V}$ = 2%), which is 8 times higher than that of graphene. The reason for above phenomena is mainly due to the hydroxyl group and the vacancy defects. Both of two factors can improve the heat transfer efficiency at the interface of adjacent GO sheets. This has been verified in previous work. For the hydroxyl group, the functionalized graphene overlaps each other. The stronger bond is increased due to the generation of hydrogen bonds between the layers, resulting in the increases of Interfacial thermal conductance [52]. For vacancy defects, the vacancy defects in adjacent graphene layer can increase their roughness and friction leading to the increasing transmission of shear phonon modes and the Interface thermal conductivity is elevated [54]. The above form also exists in GO with vacancy defects that is no hydroxyl functional group in the corresponding position of adjacent layer. Therefore, the interfacial thermal conductance can be controlled by increasing the surface functional groups and defects of GO.

**Figure 5:**
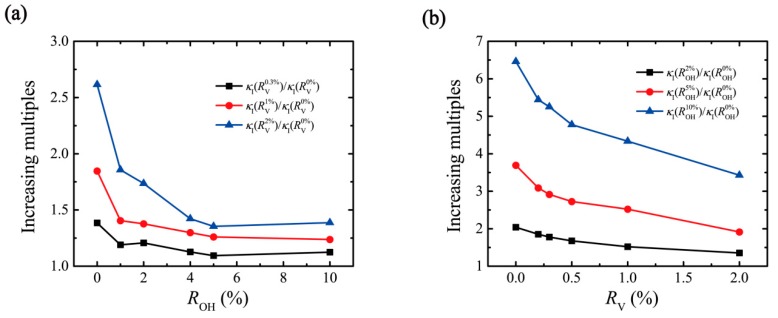
(**a**) The increasing multiples of $\kappa_{I}$ due to the degree of oxidation as a function of the vacancy defect ratio. (**b**) The increasing multiples of $\kappa_{I}$ due to vacancy defect as a function of the degree of oxidation.

**Figure 6:**
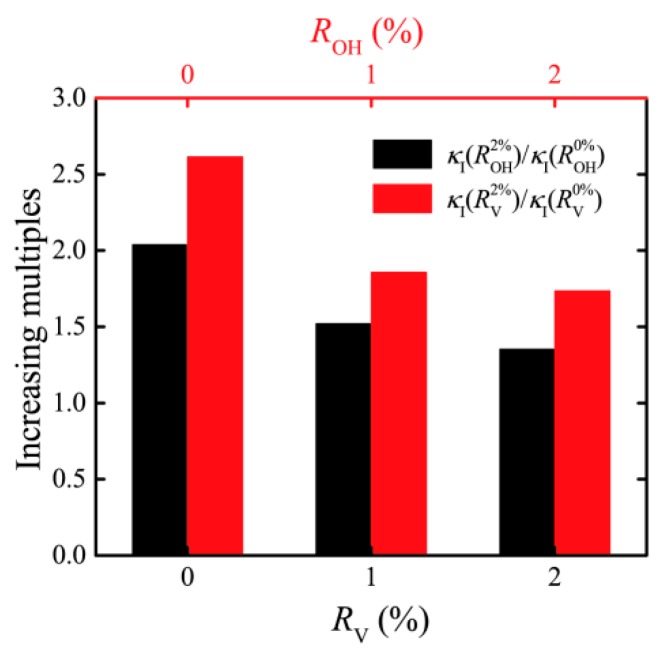
Change degree comparison of thermal conductance caused by defects and oxidation.

To further discuss the change rule of interfacial thermal conductance under the joint action of oxidation and defect, we first define increasing multiples $\kappa_{I}(R_{\mathrm{OH}}^{i})/\kappa_{I}(R_{\mathrm{OH}}^{0\%})$ as the ratio of the interfacial thermal conductance, corresponding to the degree of oxidation to the thermal conductance corresponding to the state without oxygen functional groups. Quantify the degree of thermal conductance change caused by the oxidation degree. The same definition $\kappa_{I}(R_{V}^{i})/\kappa_{I}(R_{V}^{0\%})$ quantifies the degree of thermal conductance change caused by the ratio of the vacancy defect. Figure 5a shows the ability of oxidation degree with 2%, 5%, and 10% to improve thermal conductance under different defect ratios. For the GO films without vacancy defect, the higher degree of oxidation, the stronger effect on thermal conductance. With the increase of defect ratio, the ability of thermal conductance change caused by the change of oxidation degree gradually weakened. Similarly, with the increase of oxidation degree, the ability of vacancy defect ratio change to enhance thermal conductance is also weakened (see Figure 5b). To further clarify the influence of hydroxyl and defects on thermal conductance, at the range of both oxidation degree and defect ratio are 0–2%, the improvement degrees of thermal conductance (increasing multiples) are shown in Figure 6. The height of the red column is higher than that of the black column, indicating that the vacancy defect can enhance the thermal conductance better than the hydroxyl group.

**Figure 7:**
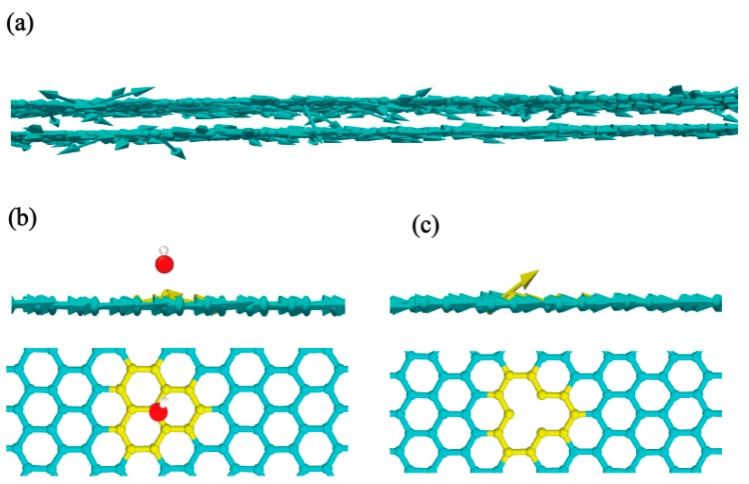
Spatial distribution of heat flux by vector arrows on each atom under nonequilibrium steady state. (**a**) The overlapping structure of GO (**b**) a hydroxyl group, and (**c**) a single vacancy defect.

To clarify the mechanism of heat transfer of GO films, the spatial distributions of the heat flux by vector arrows on each atom under nonequilibrium steady state are shown in Figure 7. The atomic heat flux is defined as $\vec{J_{i}}=e_{i}\vec{v_{i}}-S_{i}\vec{v_{i}}$, where $e_{i}$, $v_{i}$, and $S_{i}$ are the energy, velocity vector, and stress tensor of each atom, respectively [53]. It can be obtained by calculating the atomic heat flux in the reverse nonequilibrium MD simulations and the results are averaged over 2 ns in the nonequilibrium steady state. The vector arrows show the migration of the heat flux on the GO films and reflect vividly the transformation of heat flux path in the overlap model.

When a propagating phonon tries to pass through a barrier in the GO sheet, it may be scattered, and turns its original direction (in-plane direction) to the out-of-plane direction, which results in the increment of the thermal conductance. The vector arrows among the overlap structure are intensive as the increasing ratio of single vacancy and a hydroxyl group with heat flux (see Figure 7a). Especially, the influence of vacancy defect on phonon scattering is greater than that of a hydroxyl group (see Figure 7b,c), which can explain why vacancy defect has a greater effect on thermal conductance than that of the hydroxyl group at same ratio in Figure 6. 

**Figure 8:**
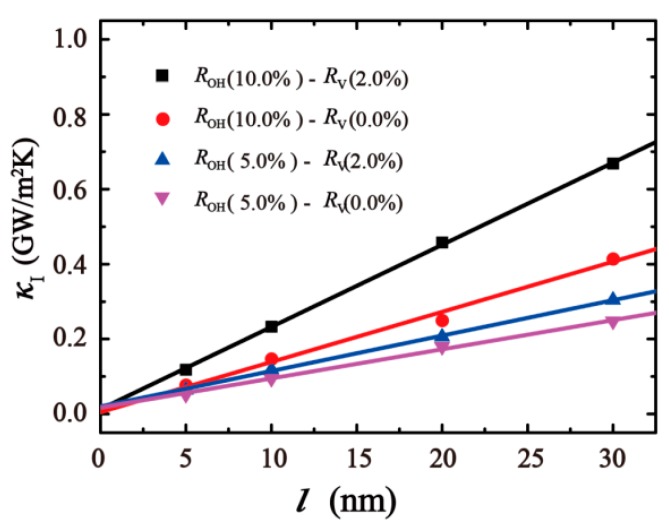
The interfacial thermal conductance $\kappa_{I}$ of four types as a function of the overlap length (5 nm, 10 nm, 20 nm, 30 nm).

To obtain the $\kappa_{\mathrm{out}}$ in Equation (11), the interfacial thermal conductance on the overlap length l is plotted in Figure 8. Four different cases are considered with overlap length l of 5, 10, 20, and 30 nm. In all the cases, the geometry of the GO sheets and their overlap area is fixed at first. Then we change the degree of oxidation and ratio of monatomic vacancy of GO films model. Four types of results are shown in Figure 6. The results show the defects of the GO sheet can improve the interfacial thermal conductance. The mechanism of functional-group heat transfer through the hydrogen bonding dominated interface suggests that the directly increasing functional density can improve the interaction for overlap structure. Meanwhile, the vacancy defects in GO sheets at the interface among the overlap structure can increase their roughness and friction, which can lead to the increasing transmission of shear phonon modes [54]. From the results, we can find the $\kappa_{I}$ depends linearly on the l and obtain $\kappa_{\mathrm{out}}^{R_{\mathrm{OH}}(10.0\%)-R_{V}(2.0\%)}$ = 21.92 × 10^6^ GWm^−3^ K^−1^, $\kappa_{\mathrm{out}}^{R_{\mathrm{OH}}(10.0\%)-R_{V}(0.0\%)}$ = 13.38 × 10^6^ GWm^−3^ K^−1^, $\kappa_{\mathrm{out}}^{R_{\mathrm{OH}}(5.0\%)-R_{V}(2.0\%)}$ = 9.45 × 106 GWm^−3^ K^−1^, $\kappa_{\mathrm{out}}^{R_{\mathrm{OH}}(5.0\%)-R_{V}(0.0\%)}$ = 7.77 × 10^6^ GWm^−3^ K^−1^ by fitting the curves in Figure 8, respectively. Within this method, $\kappa_{\mathrm{out}}$ under a varying degree of oxidation and the ratio of vacancy defect can be also obtained.

**Figure 9:**
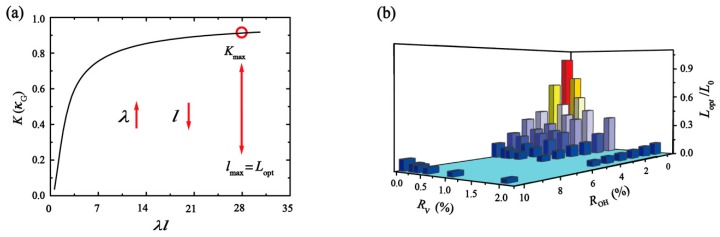
(**a**) The total thermal conductivity of GO films as a function of λl (dimensionless). The point (red symbol) is assumed as the maximum thermal conductivity in the theoretical formula. (**b**) The variation tendency of optimal overlap length for GO films with respect to the degree of oxidation and ratio of the vacancy defect.

Another key point in this work is to find the optimal overlap length $L_{\mathrm{opt}}$ of the GO films with the maximum thermal conductivity ($K_{\max}$). From Figure 9a, we can find the value of K increases with the overlap length. When the overlap length l is up to $L_{\mathrm{opt}}$, the change of K is no longer obvious and tends to converge. 

From the above discussion, $\kappa_{\mathrm{in}}$ and $\kappa_{\mathrm{out}}$ are related to the GO films structure. The efficiency of heat transfer due to the increasing ratio of functional group and single vacancy is weakened in the single-layer GO sheet (positive correlation), and enhanced in the overlap structure (negative correlation). The discrepancy between $\kappa_{\mathrm{in}}$ and $\kappa_{\mathrm{out}}$ is unified by λ in Equation (6). The larger the ratio of $R_{\mathrm{OH}}$ and $R_{V}$, the greater the value of λ. When the structure of the single-layer GO sheet is determined, that is, $\kappa_{\mathrm{in}}$ and $\kappa_{\mathrm{out}}$ are fixed, the thermal conductivity of GO films only depends on the size of overlap length to increase. To calculate the λ at different ratio of $R_{\mathrm{OH}}$ and $R_{V}$, the value of $\kappa_{\mathrm{in}}$ is obtained in previous works [40], and the value of $\kappa_{\mathrm{out}}$ is acquired by Equation (9). Then, $L_{\mathrm{opt}}$ of the structure is obtained by λl = 28. The $L_{\mathrm{opt}}$ as a function of $R_{\mathrm{OH}}$ and $R_{V}$ is shown in Figure 9b, and the benchmark values ($L_{0}$) of systems is calculated for graphene film.

When the ratio of vacancy defect is constant, the overlap length corresponding to $K_{\max}$ decrease as the increasing degree of oxidation. Analogously, overlap length corresponding to $K_{\max}$ decrease as the increasing single vacancy when the proportion of the functional group is constant. For GO films with degree of oxidation and vacancy defect ratio, the high thermal conductivity can be obtained with smaller individual GO sheet size. For example, we can initially estimate the optimal overlap length for the GO films ($R_{\mathrm{OH}}$ = 10%, $R_{V}$ = 2%) as 0.33 μm, through the following parameters, $\kappa_{\mathrm{in}}$ ~ 6.26 Wm^−1^ K^−1^, $\kappa_{\mathrm{out}}$ ~ 21.92 × 10^6^ GWm^−3^ K^−1^, λl = 28. Then, the sample size is ~ 0.66 μm. In other words, it is unnecessary to synthetic the sample size of GO films ($R_{\mathrm{OH}}$ = 10%, $R_{V}$ = 2%) beyond the size of 0.66 μm to enhance the thermal conductivity.

## 4. Conclusions

In summary, an analytical model is developed to investigate the thermal properties of overlap GO films. The influence of coupled hydroxyl group (the ratio $R_{\mathrm{OH}}$ of 0~10%), and monatomic-vacancy (the $R_{V}$ of 0~2%) on the interfacial thermal conductance of overlap GO model in the different overlapping size is studied. The results found that the thermal conductance rises under the increasing degree of oxidation and monatomic vacancy. The concept of optimal overlap length is proposed to describe the GO films with the highest thermal conductivity. The optimal length decreases with the increasing degree of oxidation and ratio of vacancy.

## Figures and Tables

**Table 1:** Interfacial thermal conductance of GO with different ratio of hydroxyl groups and vacancy defects.

	$R_{\mathrm{OH}}\;=\;0\%$	$R_{\mathrm{OH}}\;=\;1\%$	$R_{\mathrm{OH}}\;=\;2\%$	$R_{\mathrm{OH}}\;=\;3\%$	$R_{\mathrm{OH}}\;=\;4\%$	$R_{\mathrm{OH}}\;=\;5\%$	$R_{\mathrm{OH}}\;=\;10\%$
$R_{V}$ = 0.0%	0.26 *	0.42 *	0.53 *	0.75 *	0.87 *	0.96 *	1.68 *
$R_{V}$ = 0.2%	0.34 *	0.44 *	0.63 *	0.78 *	0.89 *	1.05 *	1.85 *
$R_{V}$ = 0.3%	0.36 *	0.50 *	0.64 *	0.80 *	0.98 *	1.05 *	1.89 *
$R_{V}$ = 0.5%	0.40 *	0.55 *	0.67 *	0.84 *	1.04 *	1.09 *	1.91 *
$R_{V}$ = 1.0%	0.48 *	0.59 *	0.73 *	0.89 *	1.13 *	1.21 *	2.08 *
$R_{V}$ = 2.0%	0.68 *	0.78 *	0.92 *	1.05 *	1.23 *	1.30 *	2.33 *

* units (10^8^ Wm^−2^ K^−1^).
